# CD10-Positive Extranodal Marginal Zone Lymphoma of the Thyroid Arising in Hashimoto’s Thyroiditis: A Rare Diagnostic Challenge

**DOI:** 10.7759/cureus.116738

**Published:** 2026-09-22

**Authors:** Narmeen Giacaman, Lena Nemri, Rodolfo Henrich Lobo, Lakshmi Menon

**Affiliations:** 1 Department of Internal Medicine, University of Arkansas for Medical Sciences, Little Rock, USA; 2 Department of Pathology, University of Arkansas for Medical Sciences, Little Rock, USA; 3 Department of Internal Medicine, Division of Endocrinology and Metabolism, University of Arkansas for Medical Sciences, Little Rock, USA

**Keywords:** bethesda category iii, cd10 expression, extranodal marginal zone lymphoma, extranodal marginal zone malt lymphoma, hashimoto's thyroiditis, lymphoma

## Abstract

Patients with Hashimoto’s thyroiditis (HT) have an increased risk of developing primary thyroid lymphoma (PTL), with extranodal marginal zone lymphoma (ENMZL) representing a significant subset of these cases. We present the case of a 49-year-old woman with a history of HT, Sjögren’s syndrome, and systemic lupus erythematosus who was incidentally found to have fluorodeoxyglucose (FDG)-avid thyroid nodules on PET-CT. Initial and repeat fine-needle aspiration (FNA) biopsies were either benign or indeterminate; however, flow cytometry revealed a small kappa-restricted B-cell population with dim CD10 expression. Given the persistent diagnostic uncertainty, the patient underwent total thyroidectomy, and histopathologic evaluation confirmed low-grade ENMZL with plasmacytic differentiation. Staging demonstrated localized stage IE disease. This case highlights important diagnostic challenges, including the potential for thyroid ENMZL to be missed on cytology despite ongoing clinical suspicion and the ability of rare aberrant CD10 expression in marginal zone lymphoma to mimic germinal-center-derived lymphomas.

## Introduction

Primary thyroid lymphoma (PTL) is a rare extranodal malignancy, accounting for approximately 5% of thyroid cancers and less than 3% of extranodal lymphomas [1]. It occurs most commonly in women and is strongly associated with Hashimoto’s thyroiditis (HT), with up to 78.9% of cases demonstrating evidence of underlying HT [2]. Patients typically present with a rapidly enlarging thyroid mass that may cause compressive symptoms, often mimicking progression of benign thyroid disease and complicating diagnosis.

Most PTLs are B-cell lymphomas, with diffuse large B-cell lymphoma and extranodal marginal zone lymphoma (EMZL), also known as mucosa-associated lymphoid tissue (MALT) lymphoma, representing the predominant subtypes [1]. EMZL is an indolent B-cell neoplasm arising in the setting of chronic antigenic stimulation, particularly autoimmune conditions such as HT, which promote clonal B-cell expansion and malignant transformation [3].

Immunophenotyping plays a central role in the classification of lymphoma. CD10 is a germinal center marker typically expressed in follicular lymphoma and is characteristically absent in marginal zone lymphomas. Therefore, CD10 positivity in EMZL is rare and represents a significant diagnostic pitfall, with potential for misclassification if interpreted in isolation [4].

Here we present a case of a 49-year-old woman with HT who was ultimately diagnosed with CD10-positive EMZL, a rare presentation that masked itself behind benign-appearing cytology. This case highlights the importance of integrating cytology, flow cytometry, histopathology, molecular studies, and clinical context and contributes to the limited literature on aberrant CD10 expression in EMZL.

## Case presentation

A 49-year-old woman with a history of systemic lupus erythematosus, Sjögren’s syndrome, Raynaud’s phenomenon, and HT was incidentally found to have thyroid nodules during evaluation for monoclonal gammopathy of undetermined significance (MGUS) using a PET-CT in 2023. She had no history of head or neck radiation and no family history of thyroid malignancy. She reported mild throat fullness with swallowing but denied compressive symptoms.

A PET-CT performed in July 2023 demonstrated an enlarged thyroid with focal fluorodeoxyglucose (FDG) uptake in a right thyroid nodule (standardized uptake values (SUV): 6.8). Thyroid function tests were normal, and the thyroid peroxidase antibody level was 1960 IU/mL (reference range <=9.00 IU/mL), consistent with HT. Serial thyroid ultrasounds revealed multiple right-sided nodules, including a dominant spongiform nodule measuring up to 2.7 cm and subcentimeter nodules in the left lobe. The initial fine-needle aspiration of the right superior thyroid nodule (Figure 1) showed a mixed follicular and lymphoid population and was classified as atypia of undetermined significance (Bethesda III) [5], raising concern for either benign lymphocytic infiltration associated with HT or possible lymphoma.

**Figure 1:**
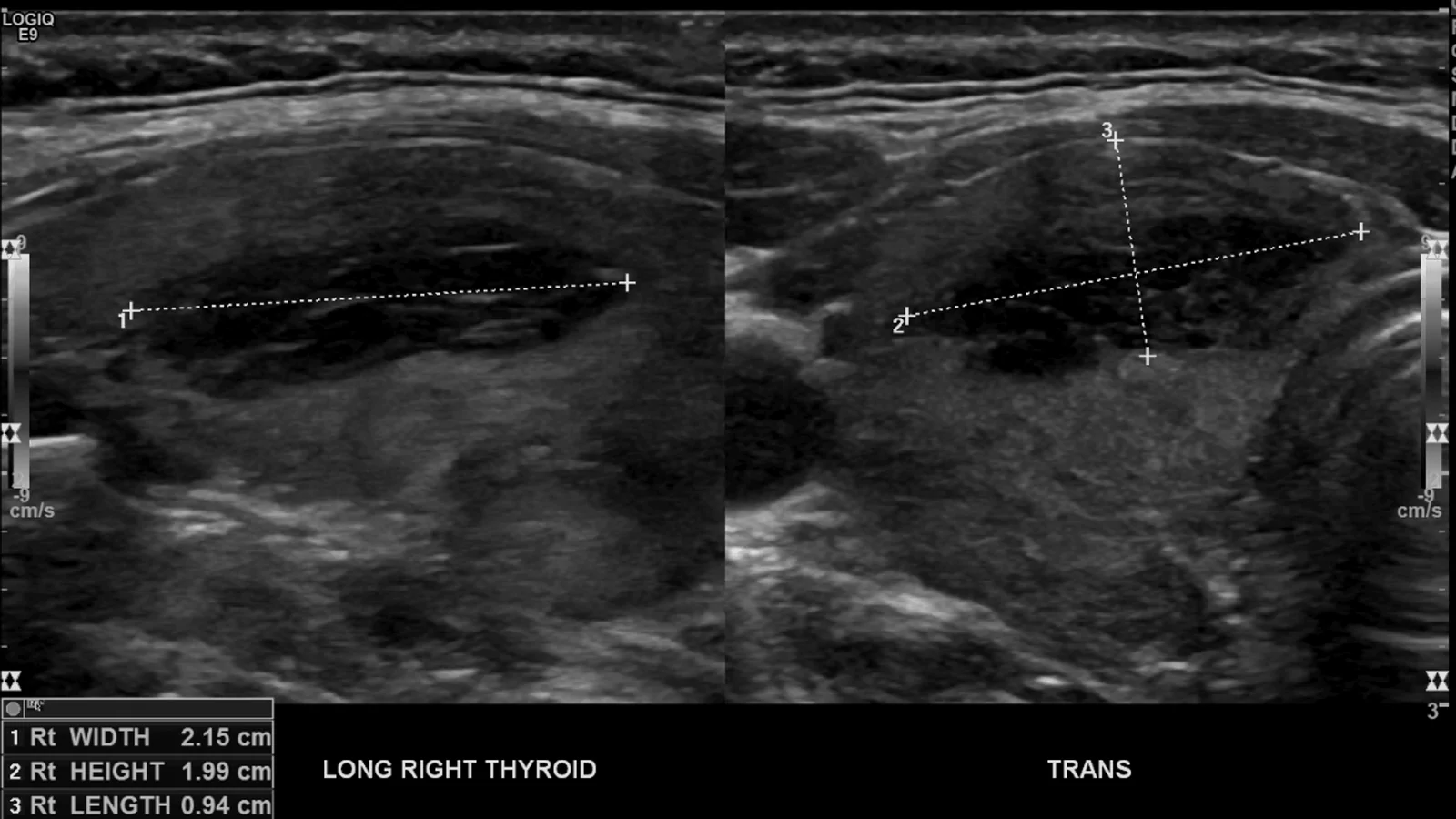
Thyroid ultrasound image showing the right superior thyroid nodule.

A repeat biopsy was subsequently performed and was negative for malignant cells (Bethesda II [5]); however, Flow cytometric analysis identified a small kappa light chain-restricted B-cell population representing 3.2% of analyzed events. This population expressed CD19 and CD20, with dim expression of CD10. No significant large-cell component was detected based on CD45 versus side scatter characteristics.

In the clinical context of HT, the significance of this kappa-restricted CD10-positive B-cell population is uncertain. The findings may reflect a small clonal B-cell proliferation or, alternatively, an atypical but reactive expansion of germinal center B cells. Correlation with cytomorphologic features and clinical findings was recommended.

Given persistent diagnostic uncertainty and patient preference, the patient underwent total thyroidectomy in January 2024. Preoperative evaluation demonstrated a healthy-appearing, clinically euthyroid female in no acute distress, without icterus or jaundice. Head and neck examination was unremarkable, with no thyromegaly or cervical lymphadenopathy. Cardiopulmonary and abdominal examinations were normal, and no lower extremity edema was noted. Laboratory studies obtained the same day showed thyroid-stimulating hormone (TSH) 3.34 µIU/mL (0.34-5.60), free thyroxine (T4) 0.73 ng/dL (0.58-1.64), and thyroid peroxidase antibody >1,000 IU/mL (≤9.00), with otherwise unremarkable metabolic and hematologic parameters. Final surgical pathology revealed a low-grade lymphoproliferative disorder with plasmacytic differentiation arising in a background of HT, with morphologic and immunophenotypic features favoring EMZL (Figures 2, 3).

**Figure 2:**
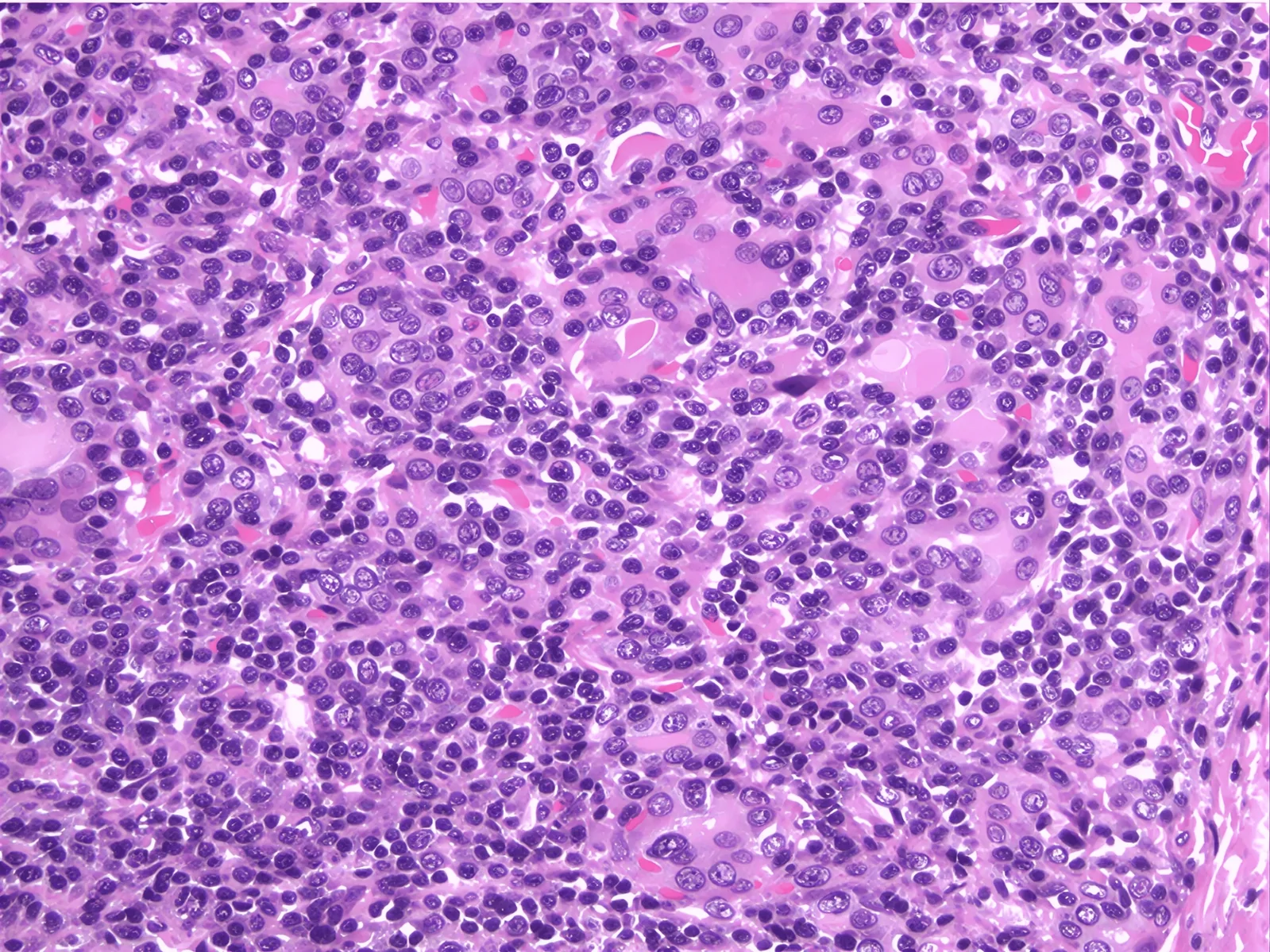
Surgical pathology specimen slide Dense lymphoplasmacytic proliferation with loss of thyroid follicles by infiltrating lymphoid cells.

**Figure 3:**
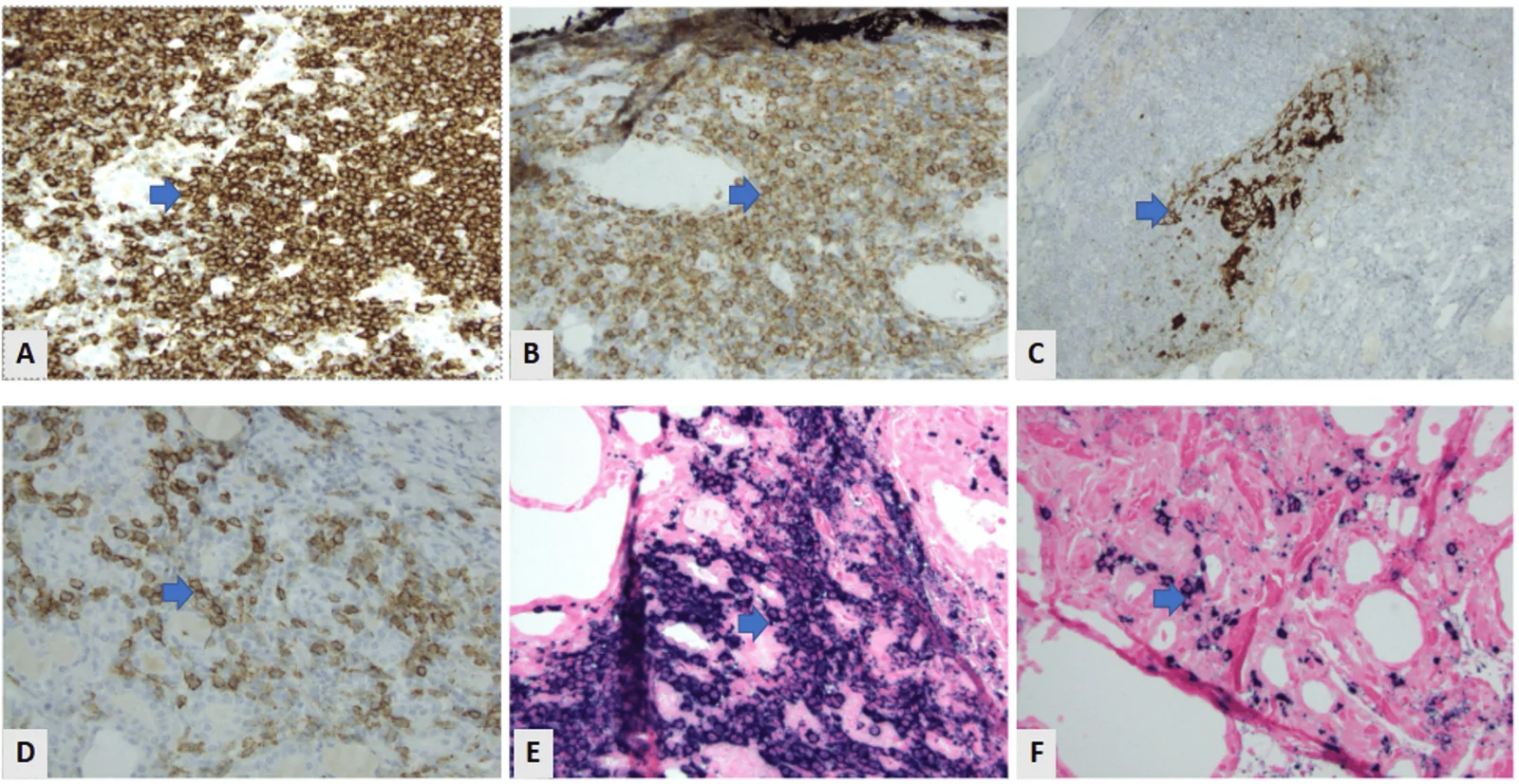
Surgical pathology immunohistochemistry staining slides A: CD20 is positive in the infiltrating B-cells; B: CD43 is aberrantly expressed in the B-cells; C: CD21 shows expanded and disrupted follicular dendritic cell meshworks; D: CD138 is positive in the infiltrating plasma cells; E–F: In situ hybridization for immunoglobulin light chains; E: Kappa light chain staining demonstrates positivity in the majority of plasma cells; F: Lambda light chain staining shows comparatively fewer positive plasma cells, supporting kappa light chain predominance.

Flow cytometry identified a small kappa-restricted B-cell population expressing CD19, CD20 (bright), and dim CD10 (Figure 4).

**Figure 4:**
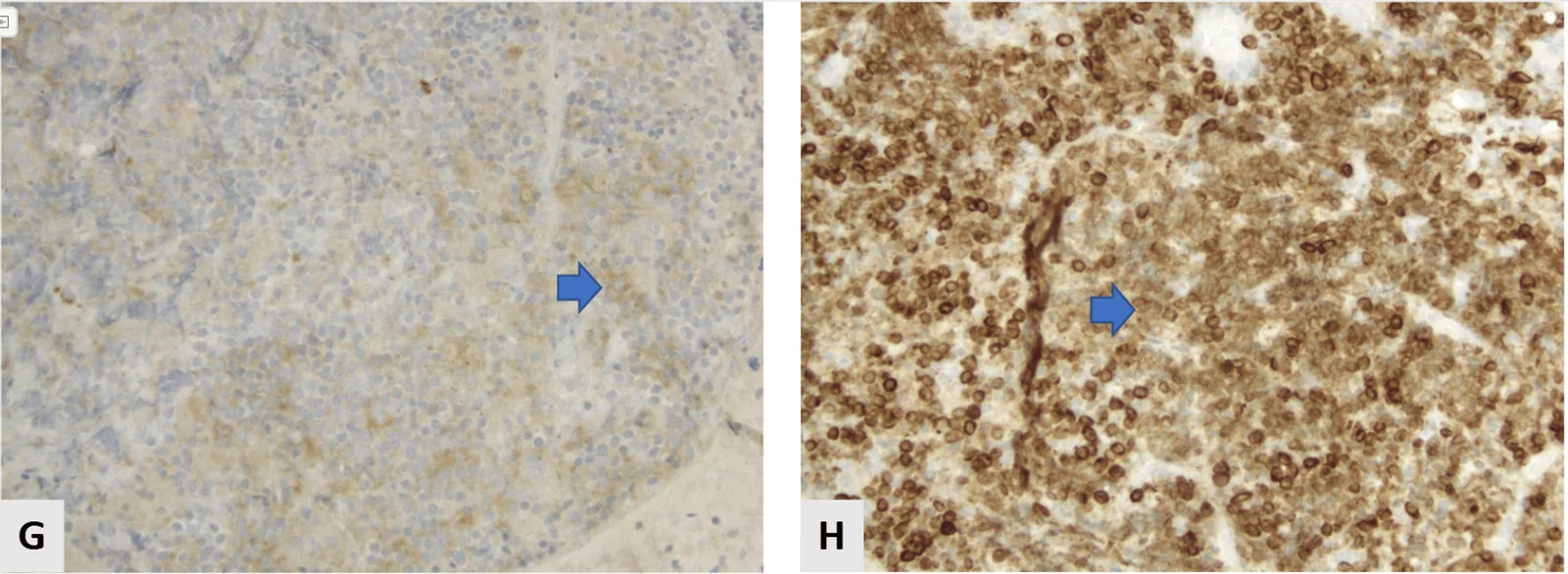
Surgical pathology immunohistochemistry staining slides G: The neoplastic B-cells are very weakly positive for CD10 (brown staining); H: The neoplastic B-cells are positive for BCL-2 (brown staining).

Staging evaluation, including bone marrow biopsy and PET-CT, showed no evidence of disseminated disease, consistent with stage IE EMZL of the thyroid. 

Postoperative PET-CT demonstrated FDG-avid soft tissue in the thyroid bed, favored to represent postsurgical changes, without FDG-avid lymphadenopathy or distant disease.

About a year post thyroidectomy, the patient palpated a left axillary node that was found to be metastatic involvement of the left axillary lymph node. Biopsy of the left axillary node was limited by scant tissue, but flow cytometry demonstrated a minute CD10-dim positive kappa monotypic B-cell population consistent with lymphoma involvement. PET-CT showed resolution of thyroid bed uptake without other definitive disease. She completed definitive radiation therapy to the left axilla and tolerated treatment well with mild nausea, fatigue, and axillary skin irritation managed conservatively.

Subsequent surveillance imaging, including serial PET-CT and CT scans of the neck, chest, abdomen, and pelvis, demonstrated no evidence of recurrent or progressive disease (Table 1).

**Table 1:** Case presentation summary FDG: fluorodeoxyglucose; SUV: standardized uptake value; FNA: fine-needle aspiration; AUS: atypia of undetermined significance; MALT: mucosa-associated lymphoid tissue; IHC: immunohistochemistry

Category	Findings
Imaging	PET-CT (July 2023): Enlarged thyroid with focal FDG-avid right thyroid nodule (SUV 6.8); Serial ultrasound: Multiple right-sided nodules, dominant spongiform nodule up to 2.7 cm, subcentimeter left-sided nodules; Post-thyroidectomy PET-CT: FDG-avid thyroid bed uptake (favored postsurgical); PET (August 2024): No FDG-avid disease; CT correlation showed ≈0.8 × 1.5 cm left axillary lymph node (biopsy-proven B-cell lymphoma); Subsequent surveillance imaging negative for progressive disease.
Cytology (FNA, October 2023)	Initial FNA: Mixed follicular and lymphoid population, Bethesda III (AUS) [5]. Repeat FNA: Negative for malignant cells (Bethesda II) [5].
Flow cytometry (thyroid FNA)	Small kappa light chain–restricted B-cell population (≈3.2%), CD19+, CD20+, dim CD10+; No large-cell population.
Surgical pathology	Total thyroidectomy (January 2024): Low-grade lymphoproliferative disorder with plasmacytic differentiation in background of Hashimoto thyroiditis; consistent with extranodal marginal zone lymphoma (MALT lymphoma).
Immunophenotype/IHC	CD20+ B-cells; aberrant CD43 expression; CD21: expanded/disrupted follicular dendritic meshworks; CD138+ plasma cells; kappa light chain predominance (kappa-restricted).
Staging	Stage IE extranodal marginal zone lymphoma of the thyroid. Bone marrow biopsy and PET-CT negative for disseminated disease.
Treatment (initial)	Total thyroidectomy (diagnostic and therapeutic).
Disease progression	≈1 year later: Left axillary lymph node involvement (CT size ≈0.8 × 1.5 cm). Biopsy limited but flow cytometry showed a minute CD10-dim kappa-restricted B-cell population consistent with lymphoma.
Treatment	Radiation therapy to left axilla: 2400 cGy in 12 fractions.
Treatment tolerance	Mild nausea, fatigue, and axillary skin irritation; managed conservatively.
Follow-up	Serial PET-CT and CT imaging: No evidence of recurrent or progressive disease after radiation therapy.

## Discussion

Marginal zone lymphomas are closely associated with autoimmune conditions such as Sjögren’s syndrome and HT [1]. They account for approximately 5%-15% of all non-Hodgkin lymphomas (NHLs) in the Western population, with EMZLs constituting around two-thirds of cases. EMZLs typically arise in extranodal tissues under chronic antigenic stimulation caused by infections or autoimmune disorders. The stomach is the most frequent site, followed by the ocular adnexa, lung, and salivary glands. Splenic marginal zone lymphomas and nodal marginal zone lymphomas make up roughly 20% and less than 10% of total cases, respectively [6].

Thyroid involvement in marginal zone lymphomas is rare and is often associated with chronic autoimmune thyroiditis [7]. HT significantly increases the risk of developing PTL, particularly ENMZL, accounting for approximately 25% of PTLs. Patients with HT have a 40-80-fold higher risk of PTL and a 67-fold increased risk of ENMZL, with incidence peaking in women around the age of 70 [1]. Therefore, ENMZL generally presents in elderly females, with peak incidence in the seventh decade and a female-to-male ratio ranging from 4:1 to 8:1 [8]. The median latency between HT and lymphoma is typically 20-30 years. Most ENMZL cases are diagnosed at early stages (I-II) and have a favorable prognosis, with a five-year disease-free survival exceeding 95%. However, transformation into high-grade lymphoma can occur, carrying a poor prognosis (five-year survival of approximately 44%). Elevated lactate dehydrogenase (LDH) or β2-microglobulin levels are often markers of transformation. Diagnosis relies heavily on histopathologic analysis due to the lack of specific clinical or biochemical markers [1].

Management of PTLs remains controversial. While complete surgical excision or radiotherapy offers excellent outcomes in localized disease, the role of aggressive debulking versus diagnostic biopsy alone is debated when complete resection is unfeasible [9]. Prognostic studies highlight that the most frequent presenting features are goiter, hoarseness, and dyspnea, with hoarseness and advanced stage (III-IV) associated with poor survival outcomes. Factors linked to poor prognosis include advanced stage, older age (>66 years), elevated serum urate, and symptoms such as hoarseness, while histologic subtype shows no significant correlation with survival [10]. 

The immunophenotypic profile of MALT lymphoma typically parallels that of marginal zone B-cells, with tumor cells expressing CD20, CD79a, and BCL2, while lacking CD5, CD10, CD23, and cyclin D1 expression. Variable expression of CD43 and CD11c may also be seen. Although no single marker is entirely specific for marginal zone lymphoma, recently proposed markers such as IRTA1 and MNDA1 may be helpful in marginal zone lymphoma diagnosis [11].

In our case, the presence of aberrant CD10 expression is particularly noteworthy. CD10 is classically associated with germinal center-derived lymphomas, including follicular lymphoma, Burkitt lymphoma, and acute lymphoblastic leukemia. Its detection in MALT lymphoma is therefore unusual and may reflect phenotypic variation or even partial transformation toward a follicular center phenotype. Rare CD10-positive marginal zone lymphomas have been documented, and distinguishing them from follicular lymphoma is essential, often requiring molecular testing such as PCR evaluation for the t (14;18) translocation [11-15].

In the case presented, a follicular growth pattern was not observed outside of the occasionally observed residual germinal centers, which were positive for CD10 and appropriately negative for BCL-2. In contrast, the neoplastic B-cell population showed weak to equivocal coexpression of CD10 by immunohistochemistry, which paralleled the dim expression of CD10 observed by flow cytometry. Additionally, the neoplastic B-cells showed clear, albeit weak, expression for BCL-2. This finding clearly separated the benign follicular structures from the lymphomatous component. 

Molecular testing was not performed in the present case, representing a limitation of this report. However, the characteristic morphologic findings, immunophenotypic profile, including aberrant CD43 expression, plasmacytic differentiation with kappa light-chain restriction, and the clinical setting of longstanding Hashimoto thyroiditis were considered sufficient to establish the diagnosis supporting an extranodal marginal zone lymphoma without the need for additional molecular confirmation.

In diagnostically challenging cases, ancillary molecular studies may provide additional support. PCR analysis for immunoglobulin heavy chain (IGH) gene rearrangements can help establish B-cell clonality when it is uncertain whether a lymphoid proliferation is reactive or neoplastic. Likewise, fluorescence in situ hybridization (FISH) for the t(14;18)(q32;q21)/IGH::BCL2 rearrangement can assist in excluding follicular lymphoma in cases with overlapping morphologic or immunophenotypic features, particularly when germinal center marker expression and overall morphology raise concern for that diagnosis. These studies are complementary to, rather than replacements for, morphologic and immunophenotypic evaluation and are most useful when the diagnosis remains equivocal after routine pathologic assessment.

In addition, this case highlights a diagnostic pitfall in PTL, demonstrating that ENMZL may remain undetected despite repeated benign fine-needle aspiration (FNA) results. Although cytology was interpreted as benign, the presence of a small kappa-restricted B-cell population on flow cytometry raised subtle concern for clonality. Definitive diagnosis was only established after total thyroidectomy, which revealed thyroid ENMZL, underscoring the limitations of cytology alone when clinical suspicion persists.

In summary, thyroid MALT lymphoma is an uncommon manifestation of marginal zone lymphomas, often associated with autoimmune thyroiditis and indolent behavior. However, atypical immunophenotypes, such as CD10 expression observed in our case, warrant careful interpretation with comprehensive pathological and molecular correlation to exclude transformation or misclassification.

## Conclusions

Thyroid ENMZL may present with benign-appearing cytology and atypical immunophenotypic features; therefore, persistent clinical concern and identification of clonal B-cell populations should prompt definitive tissue diagnosis to prevent delayed recognition of PLT. Heightened clinical vigilance is needed when cytology is benign, but clonality or clinical features raise concern of malignancy. 
